# Supplementary figures and images for: Identification of a novel base J binding protein complex involved in RNA polymerase II transcription termination in trypanosomes
Source: PLoS Genet. 2020 Feb 21;16(2):e1008390. doi: 10.1371/journal.pgen.1008390 (PMC7055916; doi:10.1371/journal.pgen.1008390)

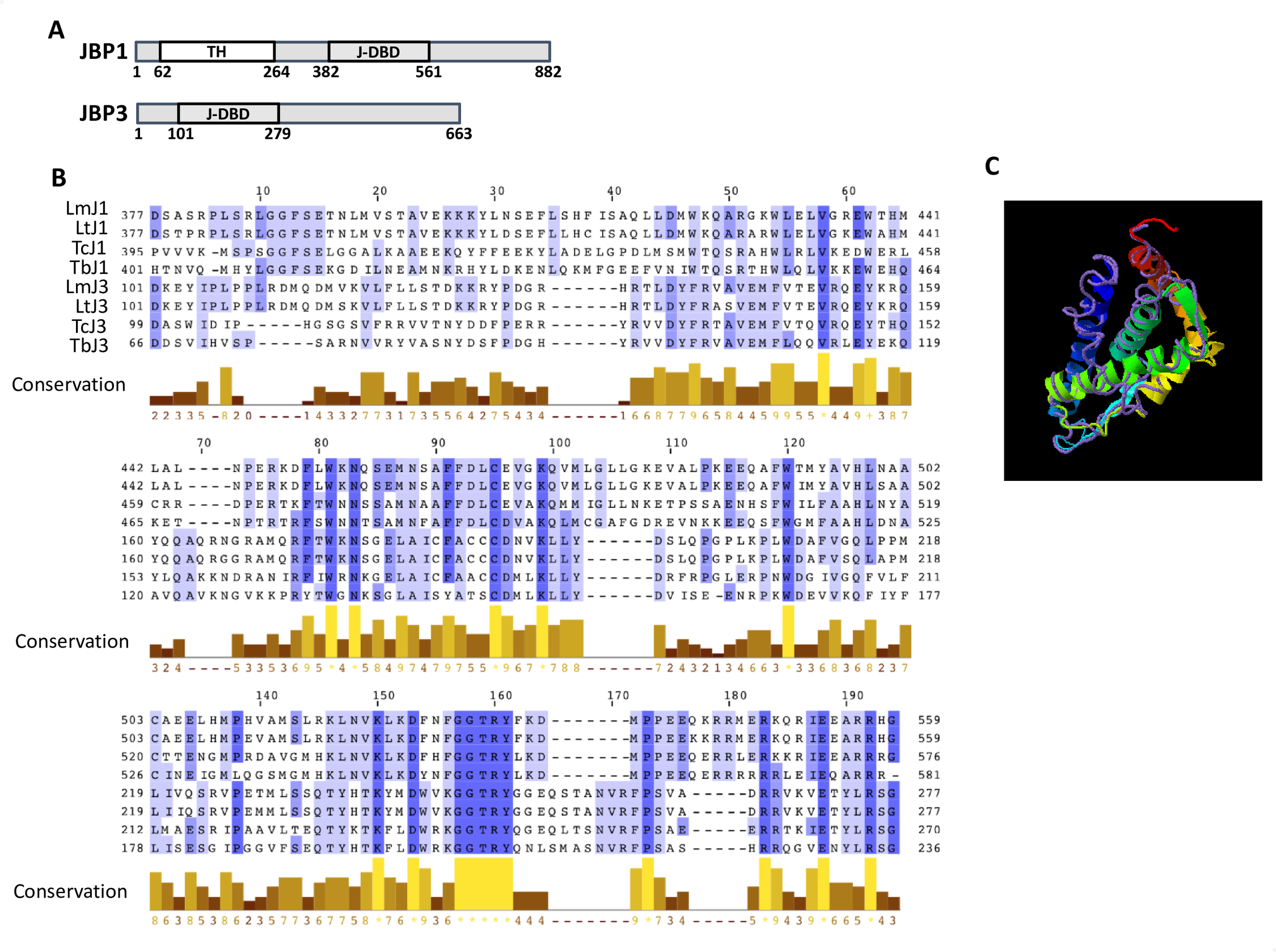

Supplement: S1 Fig — (A) Schematic representation of the structure of JBP1 and JBP3 from L. tarentolae illustrating the presence of the conserved JBD and variable C-termini. (B) A multiple sequence alignment of the JBD of JBP1 homologues from T. brucei (Tb927.11.13640), L. major (LmjF.09.1480), T. cruzi (TcCLB.506753.120), and L. tarentolae (LtaP09.1510) and the conserved region of JBP3 is shown. The sequence alignment was generated using Maft and visualized with Jalview. Identical amino acids are indicated by highlighting; >80% agreement is highlighted in mid blue and >60% in light blue. Similar amino acids are indicated by hierarchical analysis of residue conservation shown below. (C) 3D structure prediction. Using the structure of the JBD of JBP1, JBP3 was run through I-TASSER and aligns with RMSD of 1.46, Cov score of 0.774, TM-score of 0.727. In the superposition, the thick backbones are the native JBP1 JBD structure and the thin backbone is the I-TASSER model of JBP3. Blue to red runs from N- to C-terminal. (TIF) [file pgen.1008390.s001.tif]

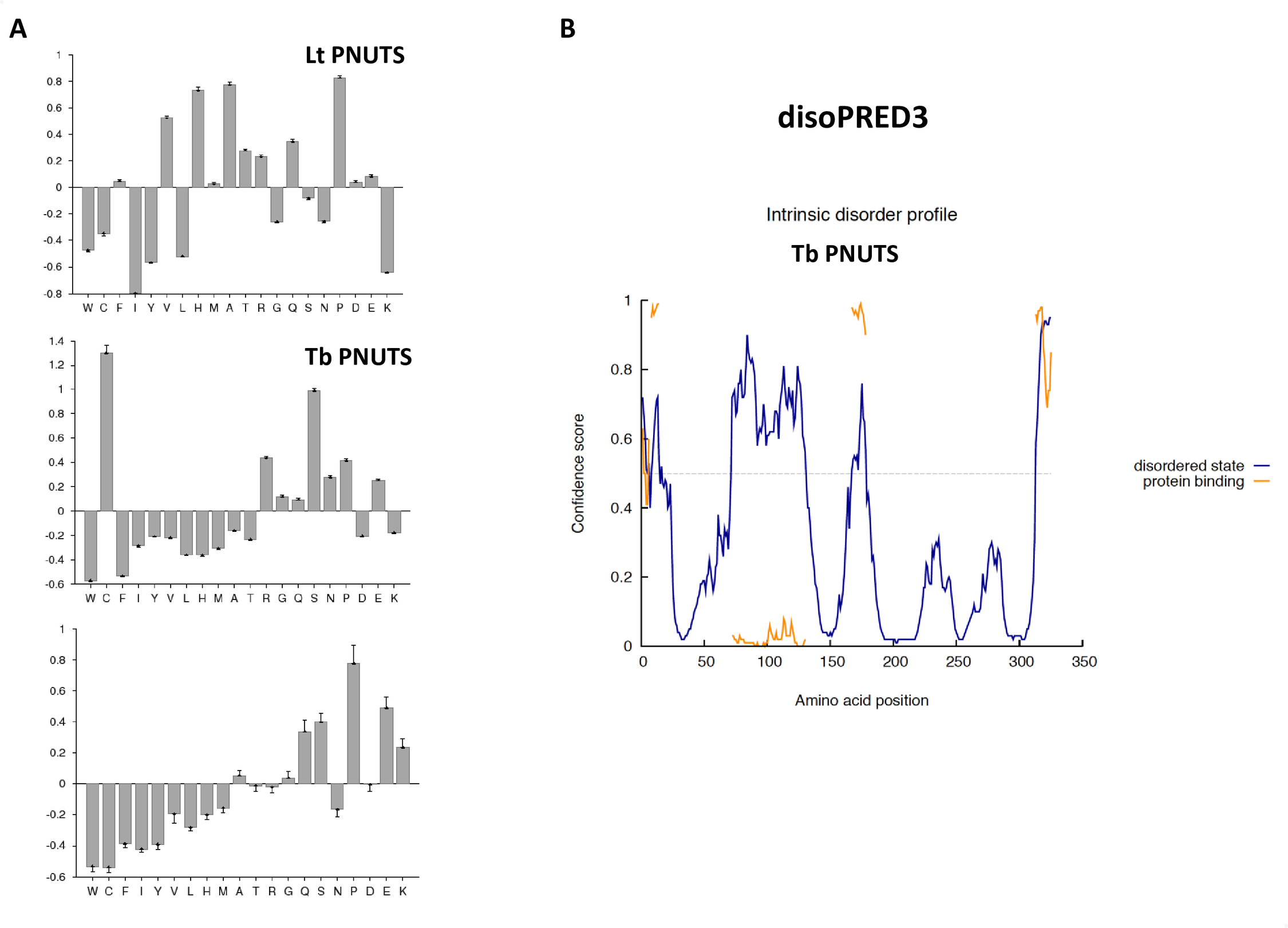

Supplement: S2 Fig — (A) Compositional profiling of Lt and Tb PNUTS showing the fractional amino acid composition in comparison with the compositional profile of typical ordered proteins. The compositional profile of typically disordered proteins from the DisProt database is shown for comparison below. (B) Analysis of TbPNUTS using the DISOPRED3 program for protein disorder prediction and for protein-binding site annotation within disordered regions. (TIF) [file pgen.1008390.s002.tif]

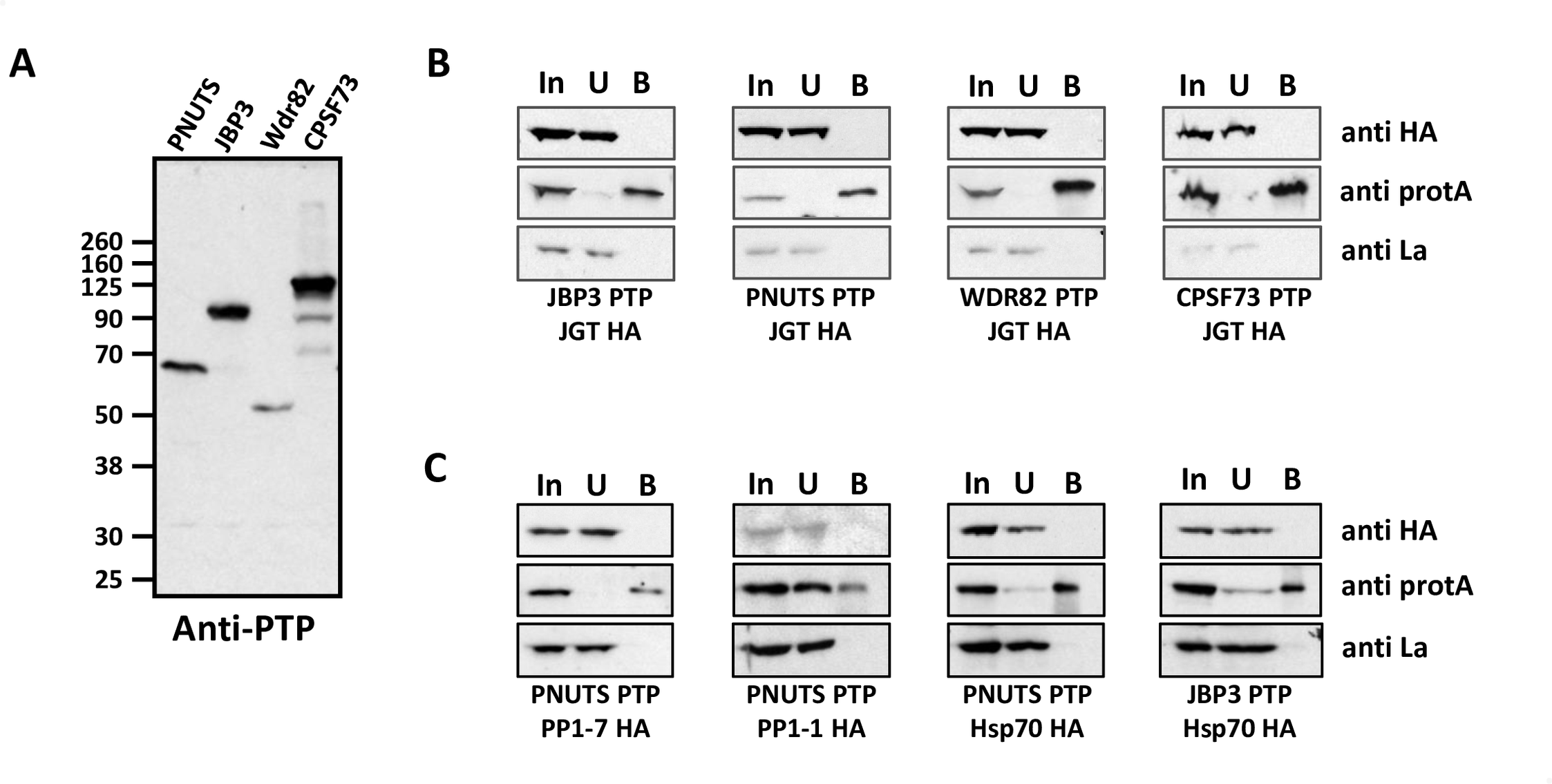

Supplement: S3 Fig — (A) Endogenously PTP tagged PNUTS, JBP3, Wdr82 and CPSF73. (A and B) Co-IP experiments as described in Fig 2A. (B) JGT is not associated with the T. brucei complex. (C) PP1-1, PP1-7 and HSP70 do not associate with PNUTS in T. brucei. (TIF) [file pgen.1008390.s003.tif]

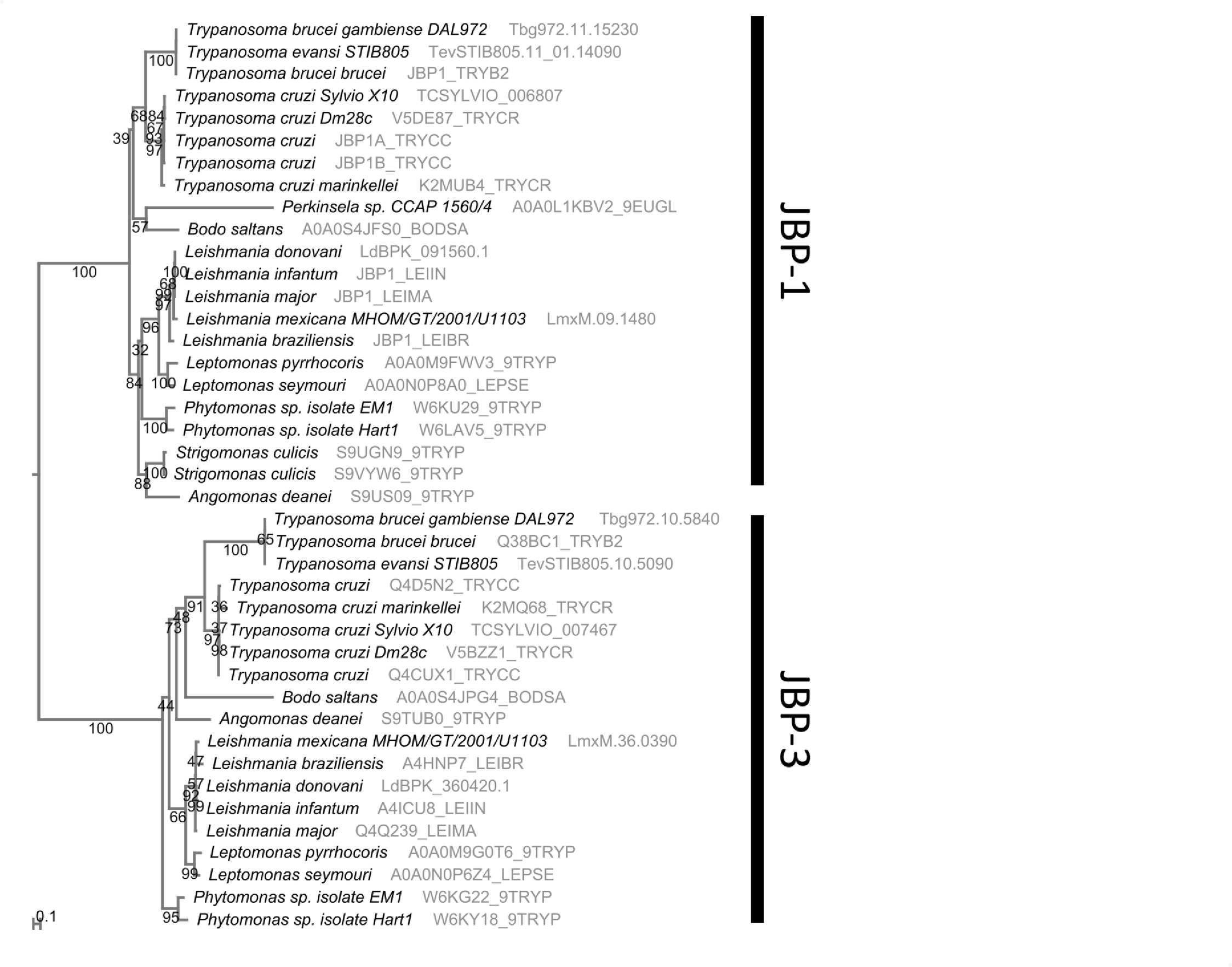

Supplement: S4 Fig — A seed alignment of JBP-1 and JBP-3 was used to iteratively search UniProtKB [132] using jackhammer [133] until convergence with an e-value cut-off of 0.01 for sequence and 0.03 for hits. Full length protein sequences from representative species were chosen and aligned using hmmalign [133] and alignancer, trimmed using trimAL [134] with the automated1 flag, and a phylogenetic tree was made using raxml [135] (options -f a -x 12345 -p 12345 -N autoMRE -m PROTGAMMAJTTF). Matching sequences were found exclusively in species within the Kinetoplastida class. The sequences separated into two distinct groups with high bootstrap support. We were able to find JBP-3 family members in all kinetoplastid genomes where JBP-1 is found, apart from Strigomonas culicis, and Perkinselia sp. which only contain JBP-1 family members. (TIF) [file pgen.1008390.s004.tif]

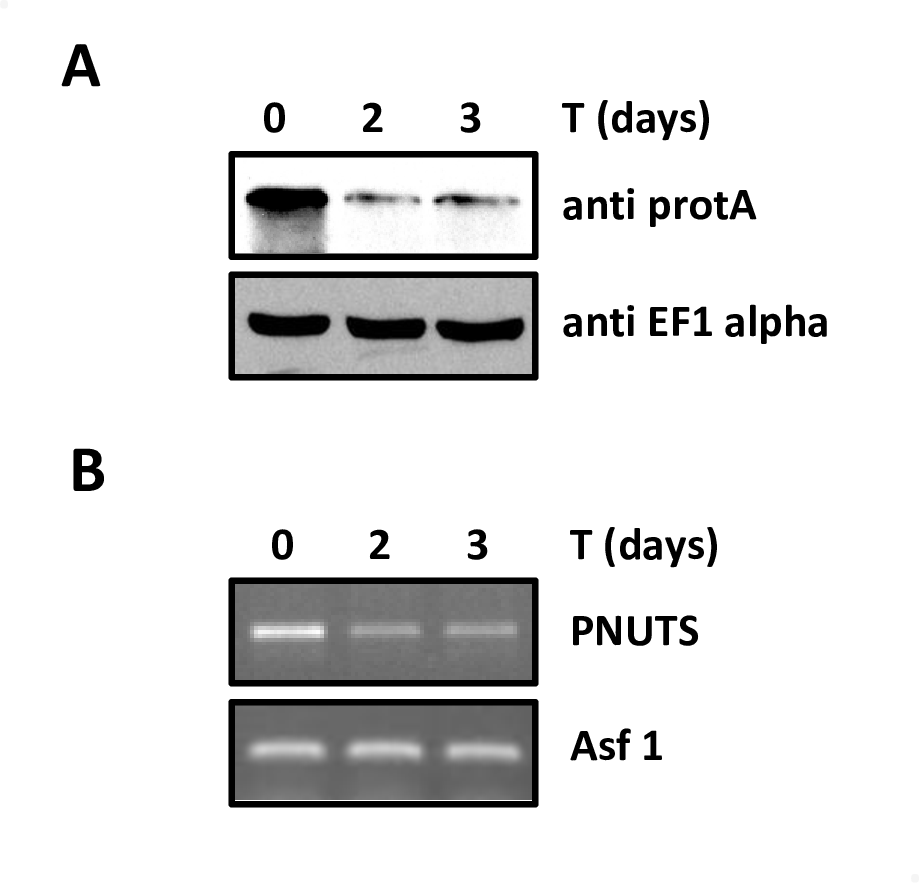

Supplement: S5 Fig — PNUTS RNAi cells with an endogenously WT PNUTS PTP tagged allele were used. Total protein and RNA was isolated after 2 and 3 days of RNAi induction and analyzed for the loss of PNUTS protein (protein A Western) and PNUTS mRNA (RT-PCR). (TIF) [file pgen.1008390.s005.tif]

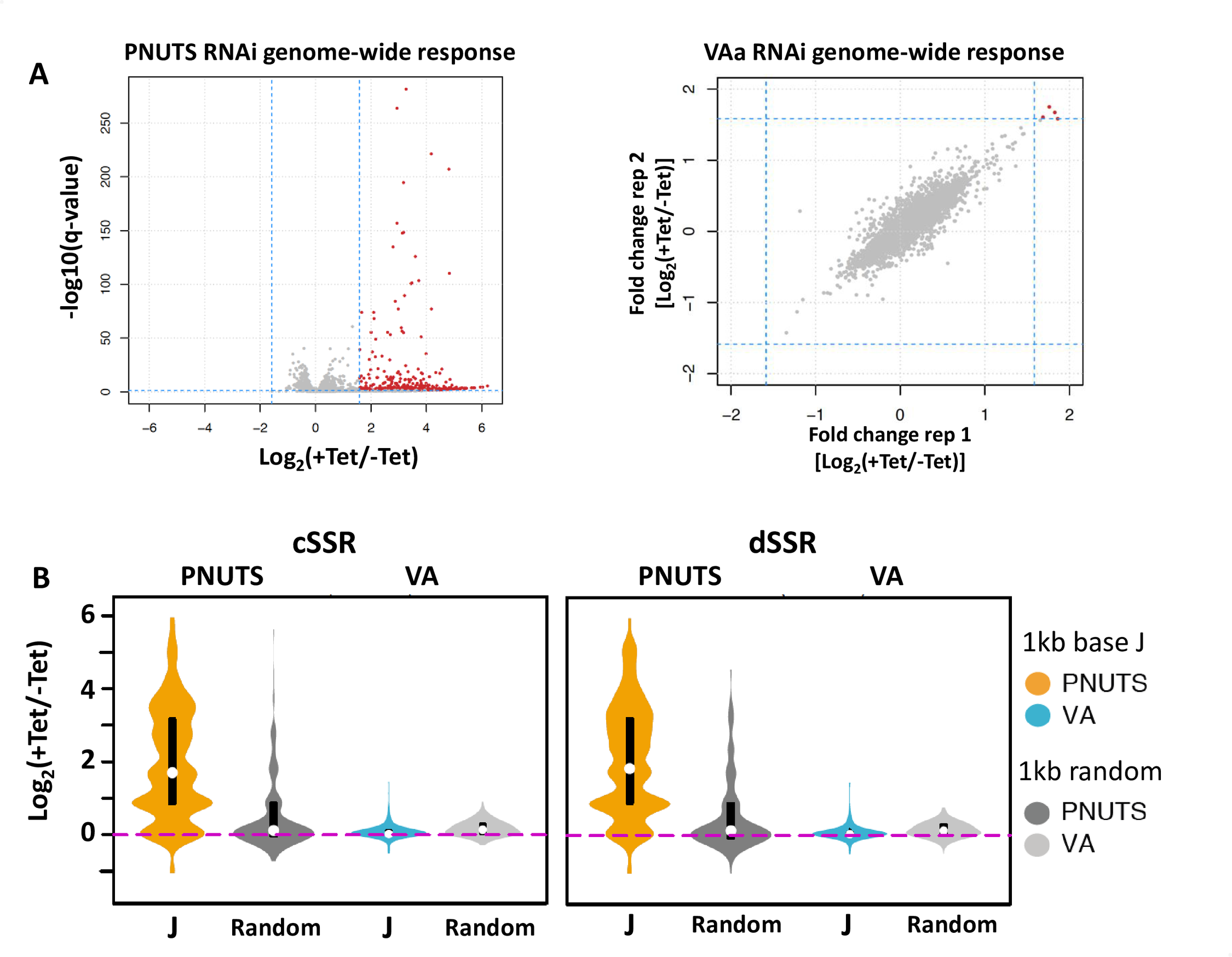

Supplement: S6 Fig — (A) Gene expression changes upon RNAi ablation of PNUTS (left) and VA a (right) are plotted. Triplicate analysis of PNUTS and duplicate of VA a. On the left, red dots represent genes with greater than 3-fold change that are also significant with a Benjamini-Hochberg FDR test correction. On the right, red dots represent genes with greater than 2-fold change after VA a ablation in both replicates. (B) Gene expression changes at cSSRs (N = 193) and dSSRs (N = 197) that are within 1kb of base J matched with same number of random locations within the genome for ablation of PNUTS and VA a. (TIF) [file pgen.1008390.s006.tif]

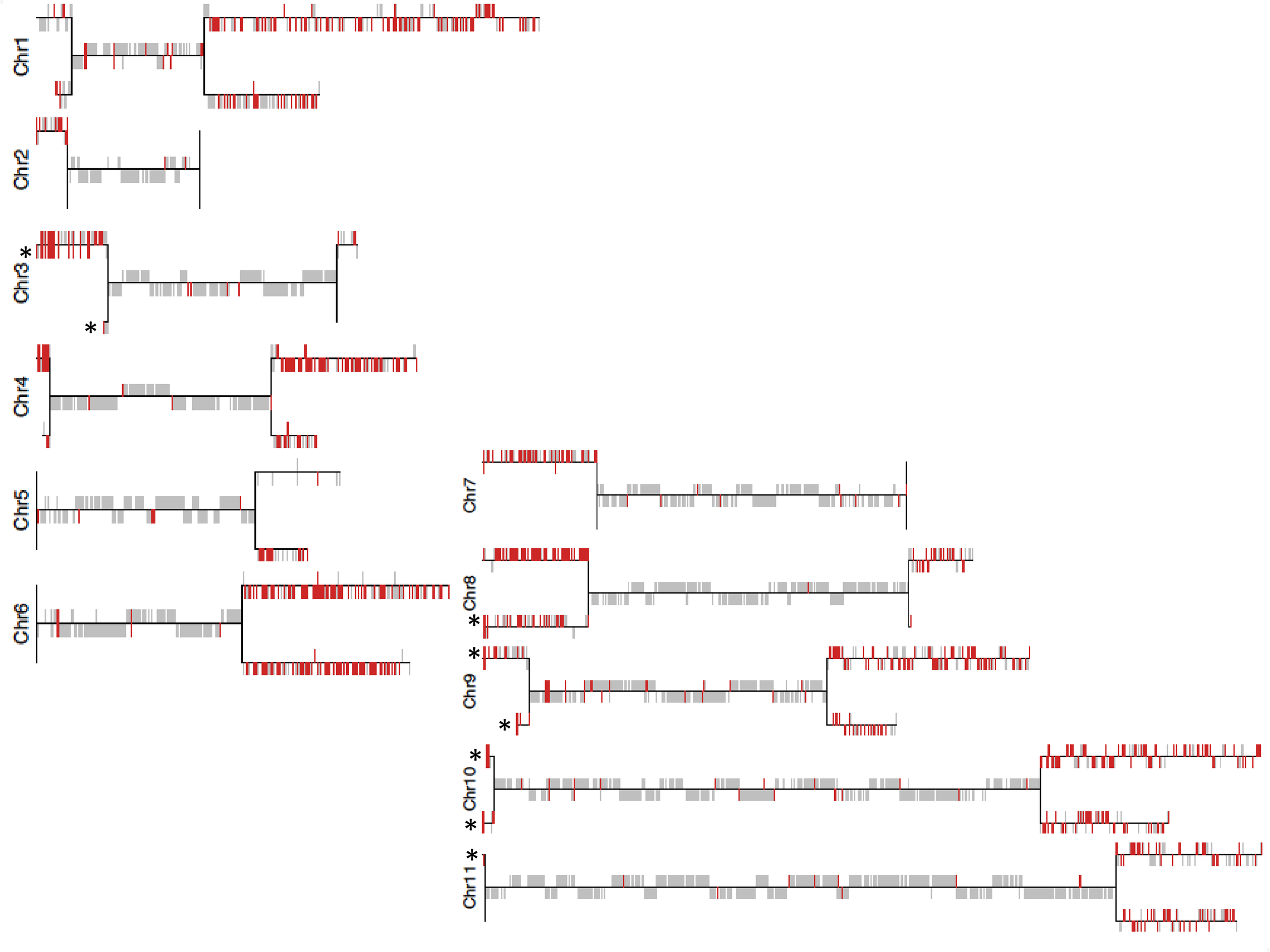

Supplement: S7 Fig — Upregulated genes (>3-fold) upon PNUTS RNAi are indicated in red in the T. brucei 427 genome assembly. Only one of the two homologous chromosomes is depicted for the homologous core regions. Both chromosomes are shown for the heterozygous subtelomeric regions containing silent VSGs. The telomeric VSG expression sites are not included in this assembly. Metacyclic-form expression sites are marked with an asterisk. (TIF) [file pgen.1008390.s007.tif]

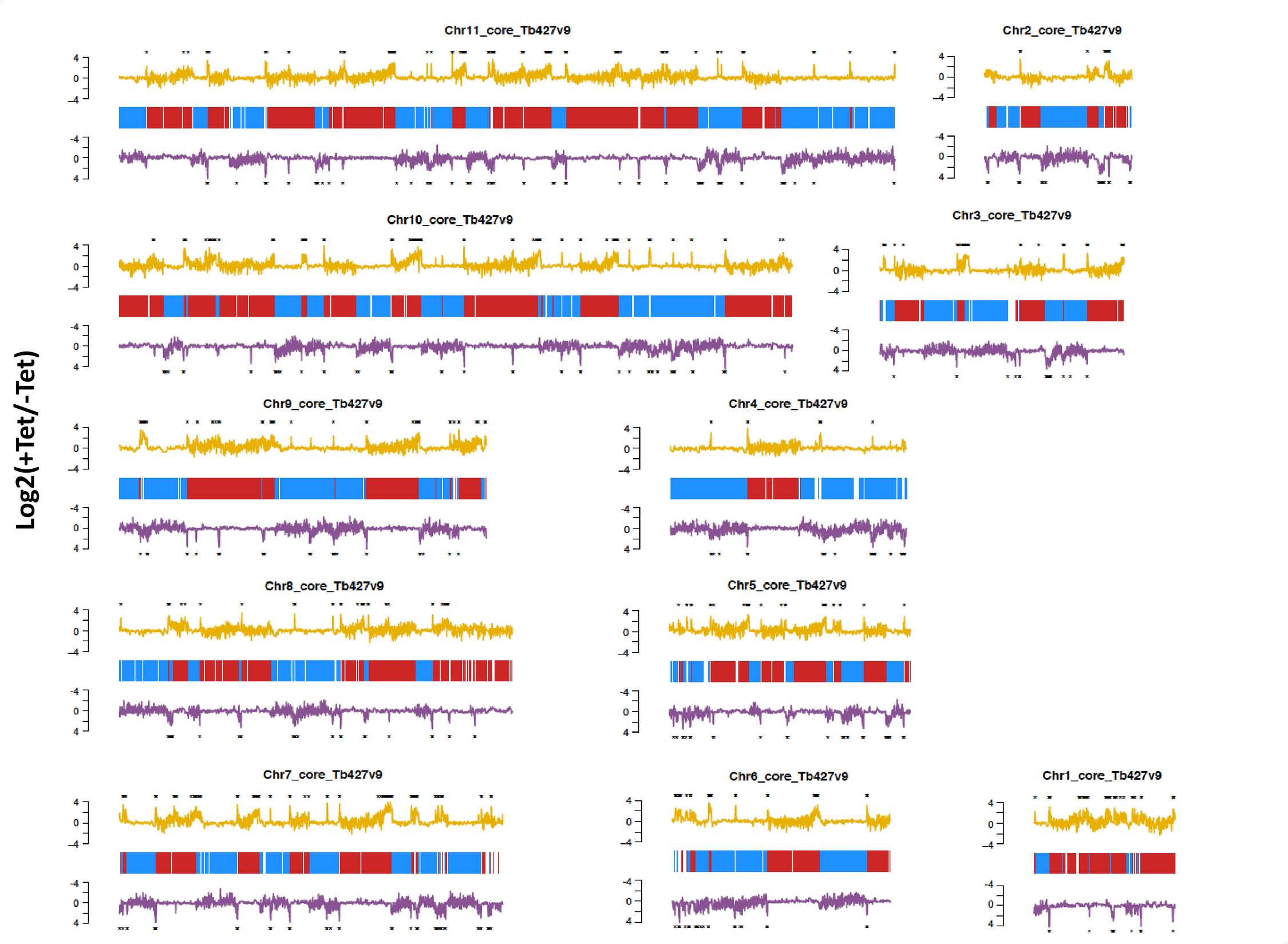

Supplement: S8 Fig — Transcription was measured by stranded RNA-seq. Fold changes comparing transcription levels between–and + tetracyclin induction of PNUTS RNAi were calculated in 200bp windows (100bp step) and plotted over the chromosome length. The core regions of the 11 chromosomes are shown. Forward (top strand) and reverse reads (bottom strand) were analyzed separately and plotted above and below the chromosome diagram, respectively. Blue genes are transcribed in reverse PTUs and red are forward PTUs. Asterix denote bins with greater than 2-fold change of expression following ablation. (TIF) [file pgen.1008390.s008.tif]

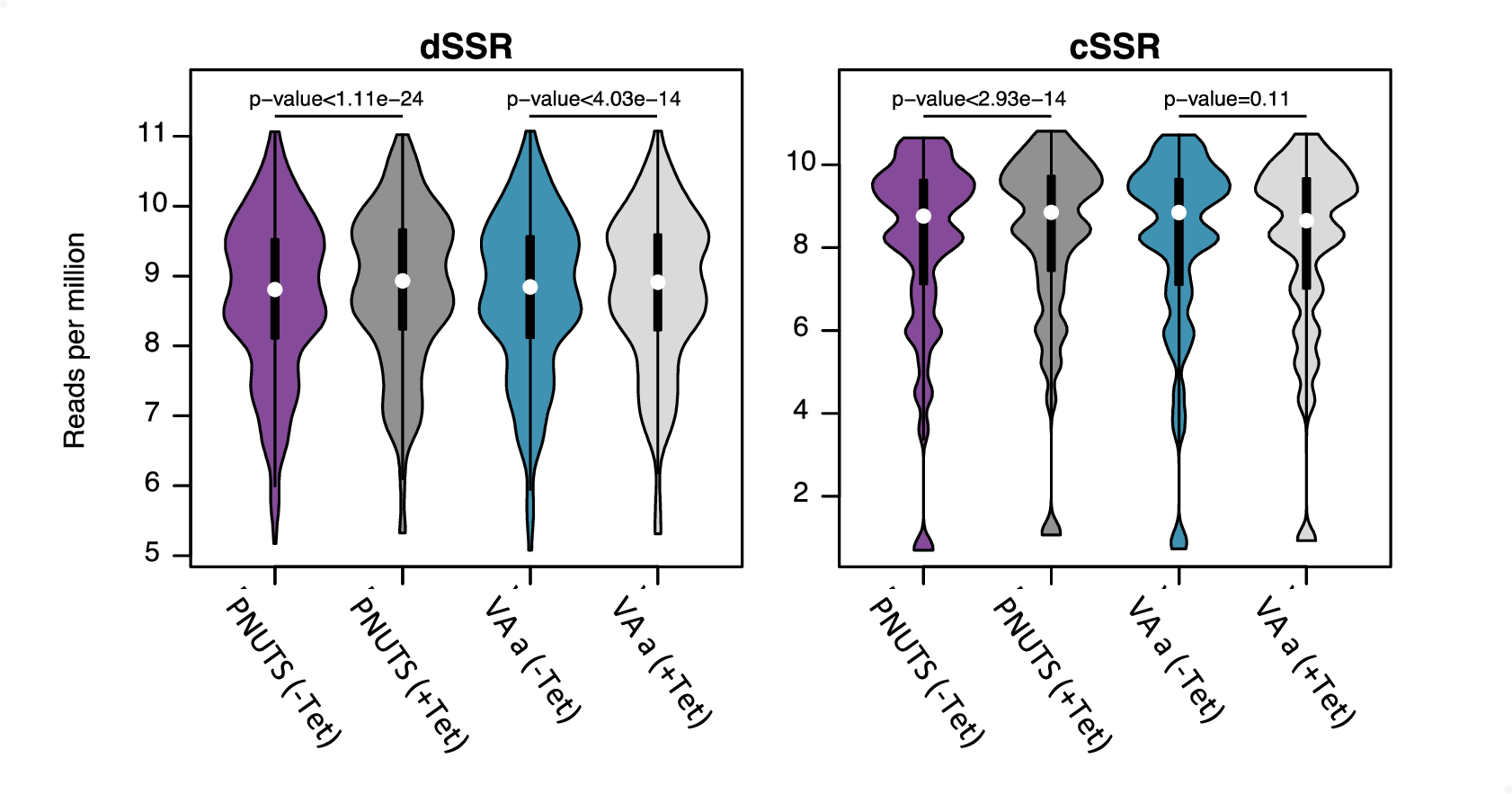

Supplement: S9 Fig — Normalized reads per million estimates were derived for dSSRs and cSSRs as the average across replicates per sample. Median values are indicated by white dots. Differences between + and–Tet were measured by a Mann-Whitney U statistical test. (TIF) [file pgen.1008390.s009.tif]

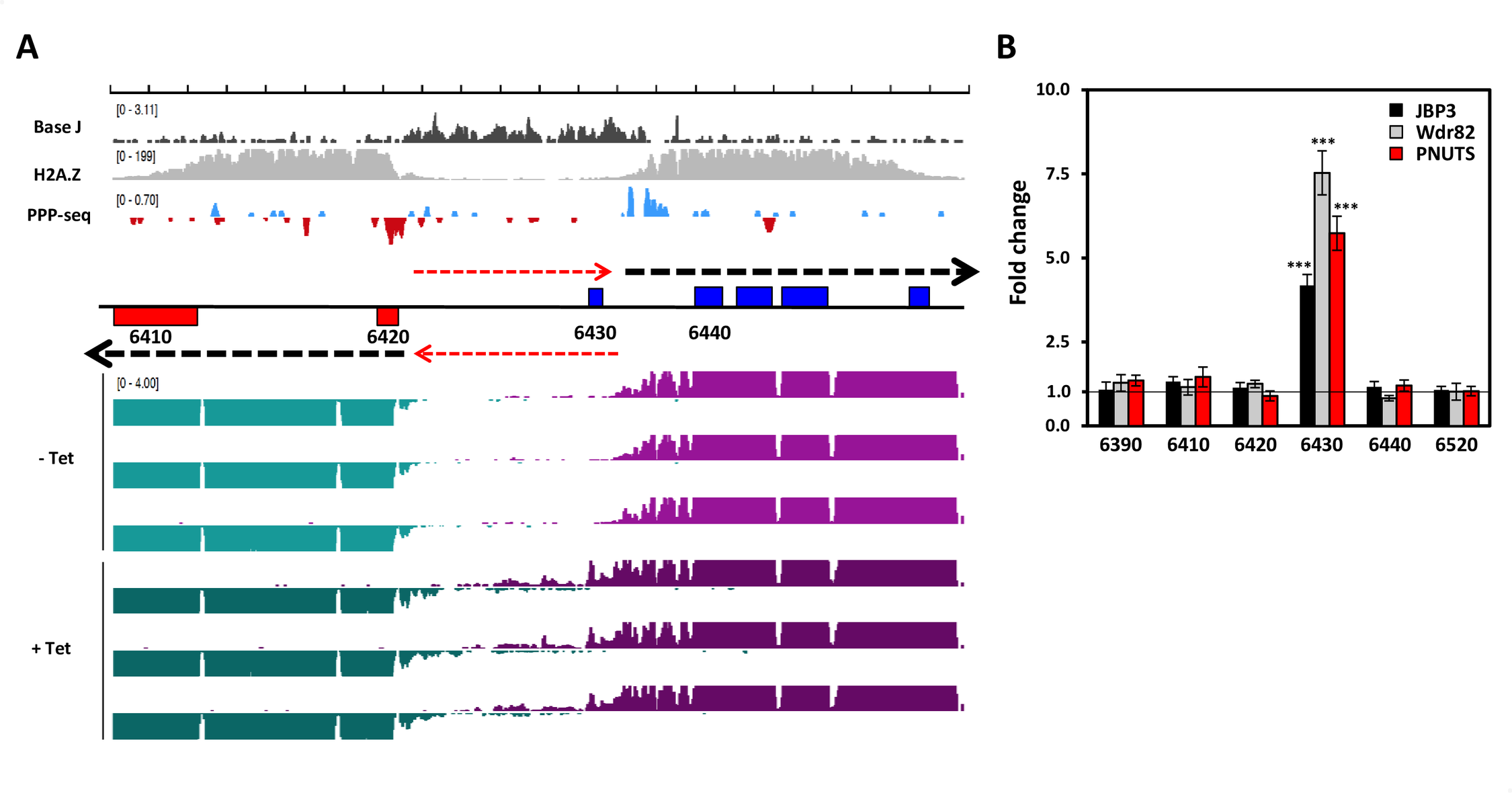

Supplement: S10 Fig — (A) Another divergent PTU region of chromosome 10 illustrating bi-directional transcription at TSSs upon TbPNUTS ablation. TSSs are denoted by PPP-seq and H2A.Z ChIP-seq enrichment in wild-type T. brucei. PPP-seq track colors: Red, reverse strand coverage; blue, forward strand coverage. RNA-seq track colors: Green, reverse strand coverage; Purple, forward strand coverage. Black arrows indicate direction of sense transcription. Red arrows indicate stimulated antisense transcription. In the case of the top strand, this ‘antisense’ transcription leads to derepression of the annotated 6430 gene (B) Confirmation of mRNA-seq transcript changes by RT-qPCR. RT-qPCR analysis was performed for the indicated genes as described in Fig 5B. RT-PCR products for gene 6430 were confirmed by DNA sequencing. (TIF) [file pgen.1008390.s010.tif]

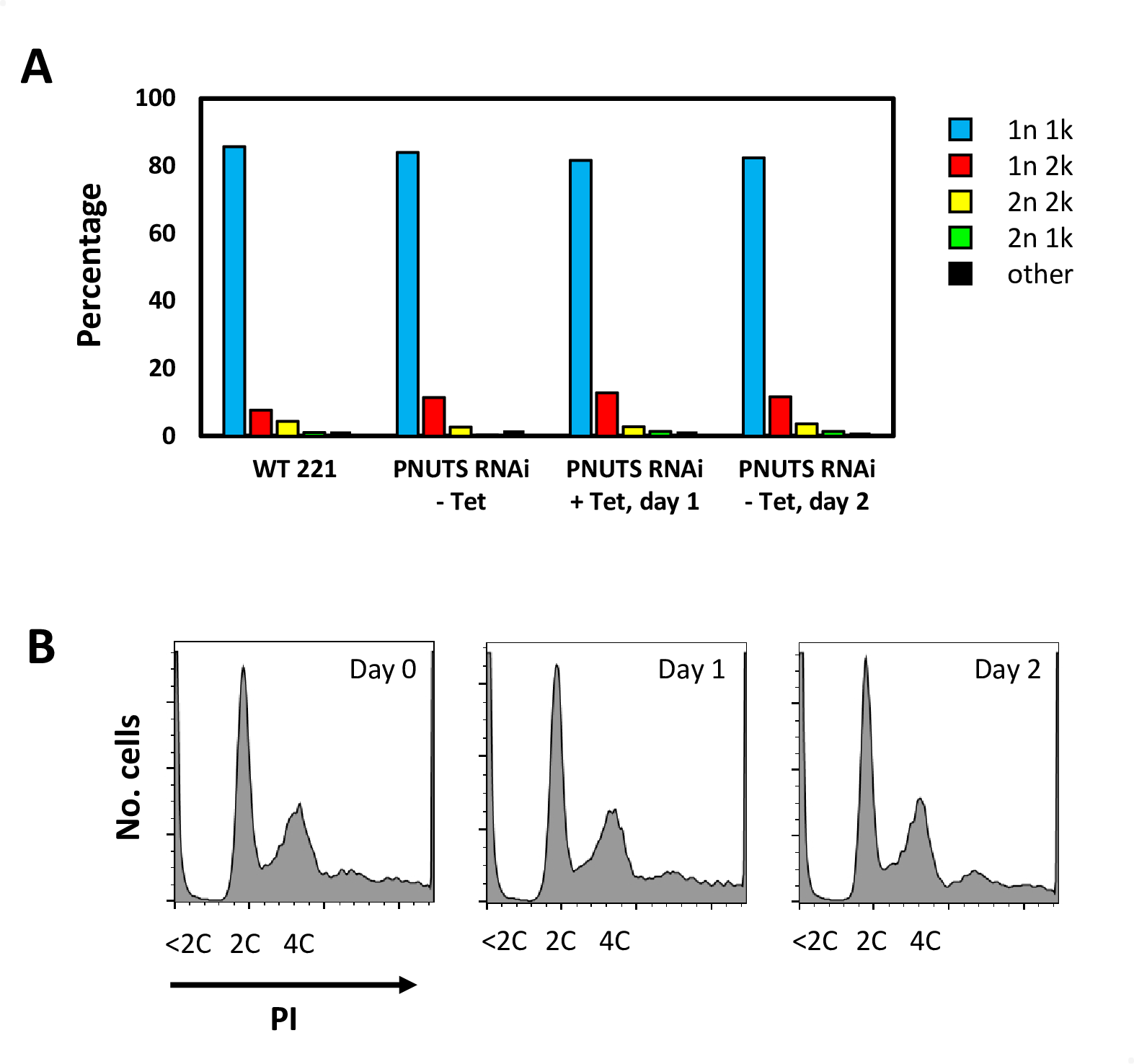

Supplement: S11 Fig — (A) Cell cycle analysis of wild-type BSF T. brucei (WT 221) and PNUTS RNAi cells–and + Tet by DAPI. (B) Cell cycle analysis using flow cytometry. TbPNUTS RNAi cells were stained with Propidium Iodide (PI) and analyzed by flow cytometry. (TIF) [file pgen.1008390.s011.tif]

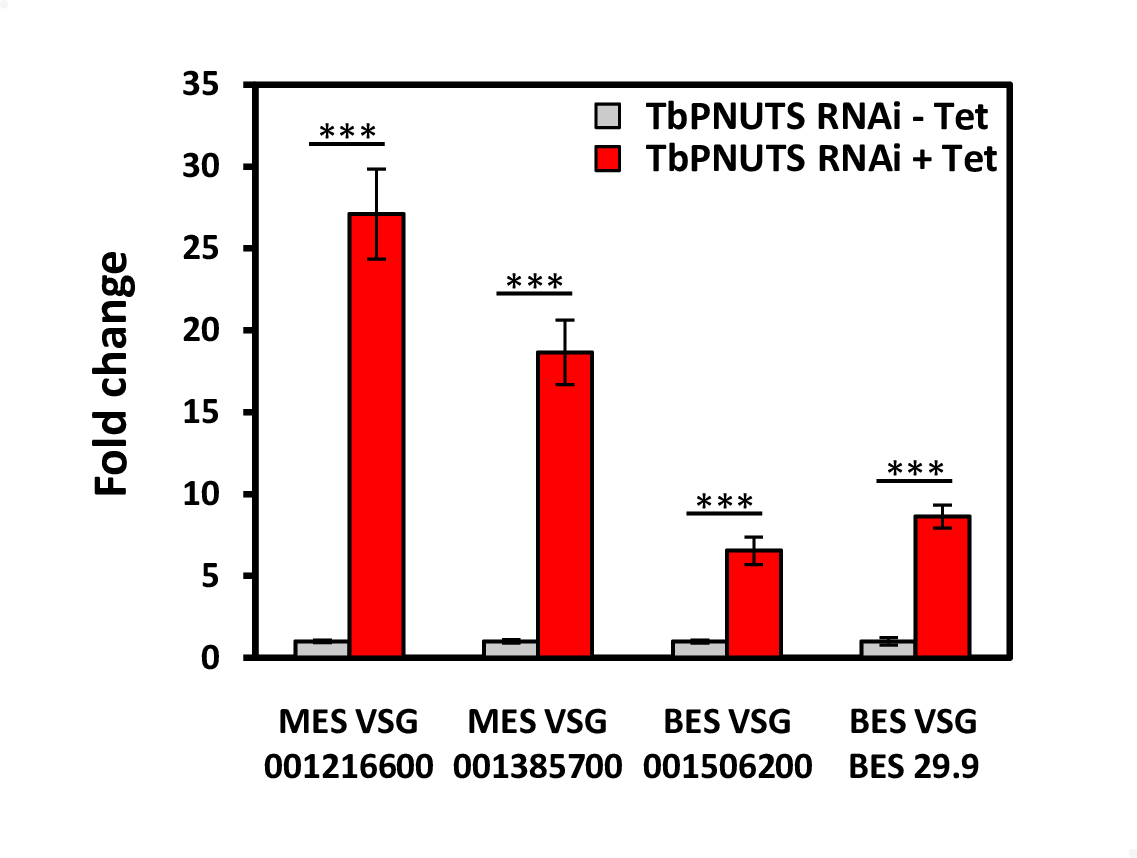

Supplement: S12 Fig — qRT-PCR analysis of MES VSG 1954, MES VSG 559, BES VSG MITat 1.1 and BES VSG MiTat 1.8 expression upon TbPNUTS ablation. Error bars indicate standard deviation from at least three experiments. P values were calculated using Student’s t test. ***, p value ≤ 0.001. All RT-PCR products were confirmed by DNA sequencing. (TIF) [file pgen.1008390.s012.tif]

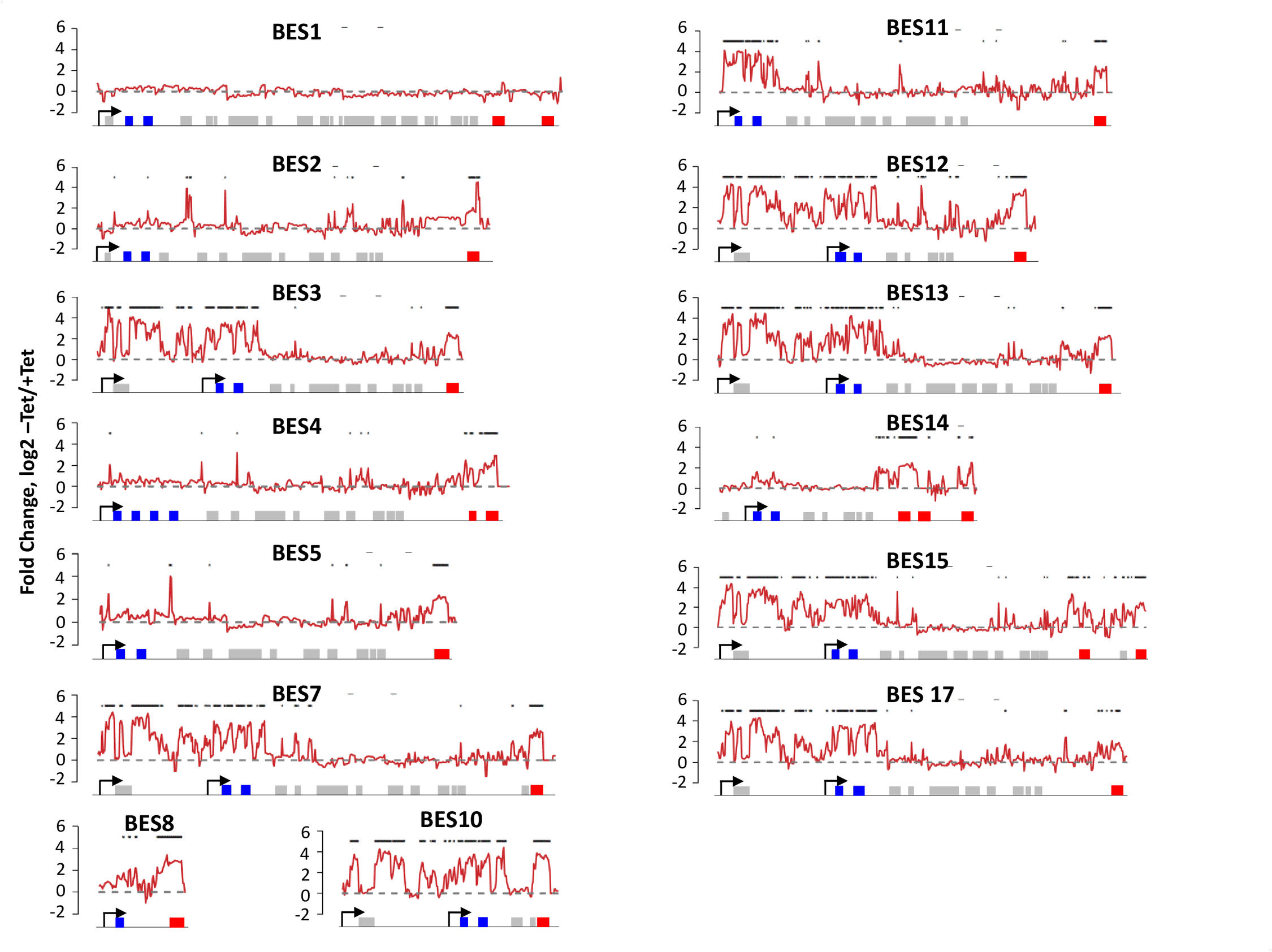

Supplement: S13 Fig — RNA-seq reads from the PNUTS RNAi were aligned to the T. brucei 427 BES sequences (14 BESs). Fold changes comparing plus and minus Tetracyclin were plotted over each BES as described in Fig 6C. (TIF) [file pgen.1008390.s013.tif]

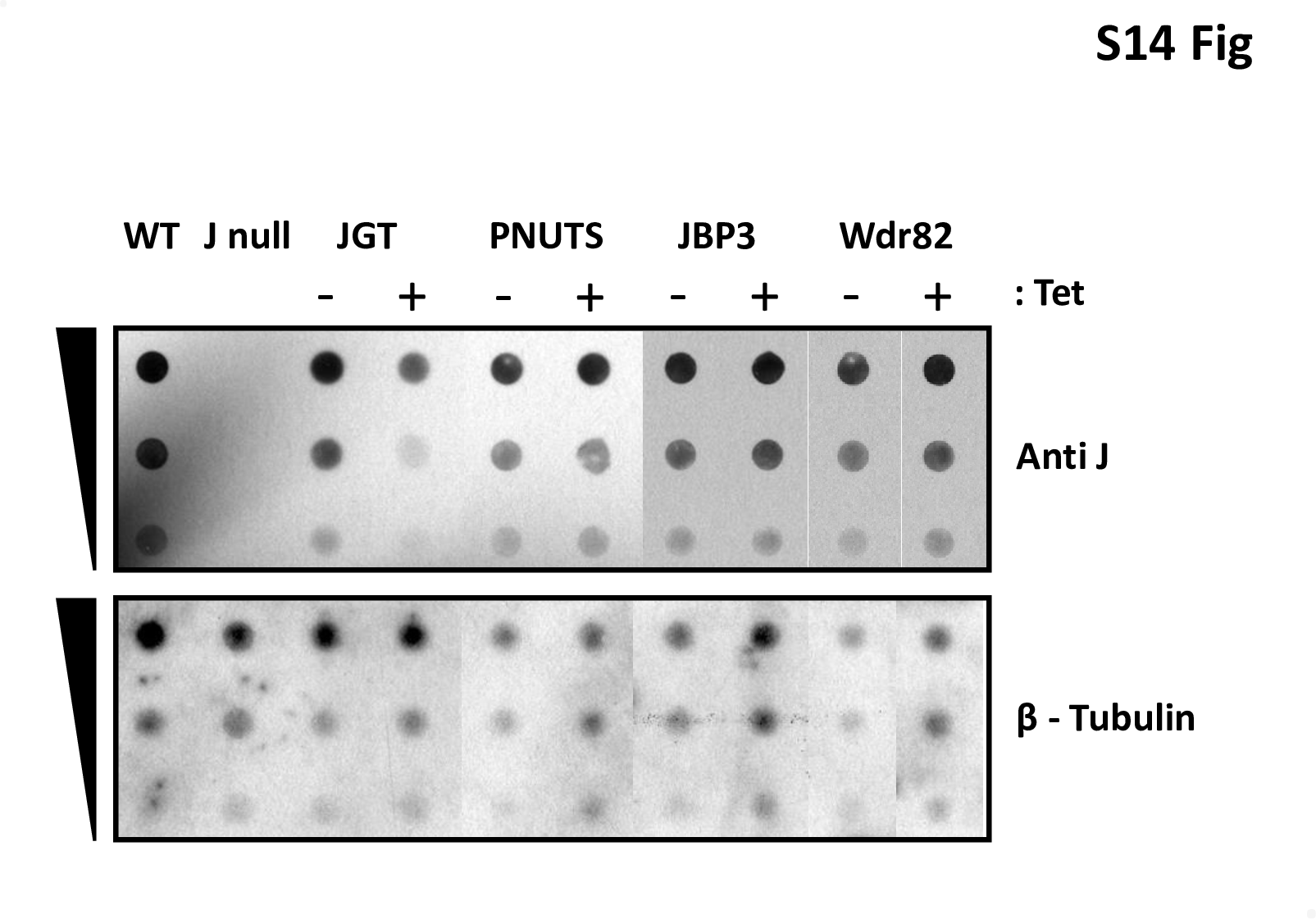

Supplement: S14 Fig — DNA was isolated from the indicated T. brucei cell lines for anti-J dot blot analysis. +Tet indicates samples from day 2 RNAi induction. Samples were 2-fold serially diluted. The same blots were hybridized with a radioactive tubulin probe to control for DNA loading. (TIF) [file pgen.1008390.s014.tif]
