# Supplementary material for: Identification of a novel base J binding protein complex involved in RNA polymerase II transcription termination in trypanosomes
Source: PLoS Genet. 2020 Feb 21;16(2):e1008390. doi: 10.1371/journal.pgen.1008390 (PMC7055916; doi:10.1371/journal.pgen.1008390)
Supplement: S1 Table — LtJGT was purified, fractionated on SDS-PAGE and proteins identified by mass spectrometry as in Table 1. Complete list of proteins identified in the JGT and the WT negative control purifications is shown. Proteins that were enriched at least 40-fold (based on PSMs) compared to the negative control purification are highlighted. (DOCX) [file pgen.1008390.s015.docx]

S1 Table: JGT purification and gel based MS analysis in *L. tarentolae*

**JGT**

**WT**

| Accession | Annotation | MW | Score | Peptides | Coverage | PSMs | Score | Peptides | Coverage | PSMs |
| --- | --- | --- | --- | --- | --- | --- | --- | --- | --- | --- |
| LtaP36.2450 | JGT | 101.3 | 37058.3 | 60 | 63.9 | 3625 | 3957.3 | 10 | 11.6 | 75 |
| LtaP33.1440 | PNUTS | 28.6 | 3698.4 | 8 | 42.8 | 61 | 0 | 0 | 0 | 0 |
| LtaP32.3990 | Wdr82 | 41.5 | 6721.1 | 14 | 36.4 | 185 | 215.8 | 1 | 2.6 | 1 |
| LtaP36.0380 | JBP3 | 73.9 | 5328.7 | 13 | 16.7 | 128 | 0 | 0 | 0 | 0 |
| LtaP15.0230 | PP1 | 42.3 | 4084.1 | 9 | 20.6 | 114 | 0 | 0 | 0 | 0 |
| LtaP18.0230 | RNA-binding protein, putative | 43.5 | 1172.3 | 3 | 7.1 | 8 | 377.6 | 1 | 3.4 | 2 |
| LtaP28.1290 | Hypothetical protein, conserved | 50.1 | 1066.7 | 3 | 7.9 | 12 | 0 | 0 | 0 | 0 |
| LtaP28.1740 | Sulfate transporter-like protein | 217.8 | 470.3 | 2 | 0.9 | 2 | 575.4 | 2 | 0.9 | 14 |
| LtaP27.1870 | Hypothetical protein, unknown function  (GO function: transcription initiation) | 80.0 | 218.4 | 1 | 1.1 | 8 | 824.3 | 3 | 3.9 | 44 |
| LtaP29.0900 | High mobility group protein homolog tdp-1, putative | 33.9 | 806.7 | 3 | 8.3 | 5 | 0 | 0 | 0 | 0 |
| LtaP20.0260 | Hypothetical protein, conserved | 541.2 | 520.1 | 2 | 0.3 | 18 | 695.7 | 3 | 0.5 | 16 |
