# Supplementary material for: Identification of a novel base J binding protein complex involved in RNA polymerase II transcription termination in trypanosomes
Source: PLoS Genet. 2020 Feb 21;16(2):e1008390. doi: 10.1371/journal.pgen.1008390 (PMC7055916; doi:10.1371/journal.pgen.1008390)
Supplement: S2 Table — LtJGT, LtJBP3 and LtWdr82 proteins were purified and proteins in the soluble fraction identified by shotgun proteomics. Proteins that were enriched at least 10-fold (based on PSMs) compared to the negative control purification are highlighted. Pep: Peptides; Cov: Coverage. (DOCX) [file pgen.1008390.s016.docx]

S2 Table: Mass spectrometric identification of JGT, JBP3 and Wdr82 purification products

**WT**

**JBP3**

**JGT**

**Wdr82**

| Accession | Annotation | MW | Score | # Pep | % Cov | PSMs | Score | # Pep | % Cov | PSMs | Score | # Pep | % Cov | PSMs | Score | # Pep | % Cov | PSMs |
| --- | --- | --- | --- | --- | --- | --- | --- | --- | --- | --- | --- | --- | --- | --- | --- | --- | --- | --- |
| LtaP36.2450 | JGT | 101.3 | 154.5 | 24 | 36.6 | 396 | 63.5 | 14 | 24.4 | 67 | 34.1 | 9 | 16.3 | 36 | 0 | 0 | 0 | 0 |
| LtaP33.1440 | PNUTS | 28.6 | 24.3 | 6 | 35.2 | 35 | 40.5 | 9 | 39.8 | 127 | 53.1 | 10 | 46.6 | 310 | 0 | 0 | 0 | 0 |
| LtaP32.3990 | Wdr82 | 41.5 | 10.3 | 4 | 12.4 | 14 | 29.6 | 8 | 22.0 | 162 | 38.6 | 9 | 26.6 | 322 | 1.1 | 1 | 3.4 | 1 |
| LtaP36.0380 | JBP3 | 73.9 | 5.8 | 2 | 4.2 | 10 | 34.1 | 7 | 20.4 | 117 | 21.5 | 6 | 13.3 | 56 | 0 | 0 | 0 | 0 |
| LtaP15.0230 | PP1 | 42.3 | 0 | 0 | 0 | 0 | 12.3 | 4 | 8.6 | 18 | 28.0 | 6 | 16.3 | 56 | 0 | 0 | 0 | 0 |
| LtaP33.0890 | unspecified product | 13.4 | 8.9 | 2 | 16.4 | 12 | 12.2 | 3 | 16.4 | 18 | 9.3 | 2 | 16.4 | 14 | 13.5 | 3 | 16.4 | 31 |
| LtaP34.3340 | Dna topoisomerase IB, large subunit | 74.0 | 5.8 | 1 | 3.0 | 4 | 4.9 | 1 | 3.0 | 4 | 3.4 | 1 | 3.0 | 4 | 4 | 1 | 3.0 | 8 |
