# Supplementary material for: Identification of a novel base J binding protein complex involved in RNA polymerase II transcription termination in trypanosomes
Source: PLoS Genet. 2020 Feb 21;16(2):e1008390. doi: 10.1371/journal.pgen.1008390 (PMC7055916; doi:10.1371/journal.pgen.1008390)
Supplement: S3 Table — TbPNUTS was purified and proteins in the soluble fraction identified by shotgun proteomics. List of PNUTS-PTP co-purified proteins, identified at 1% FDR by LC-MS/MS, with 10 or more PSMs are shown. Proteins that are enriched at least 40-fold compared to the negative control purification of wildtype extract and a score of at least 100 are highlighted. Included are proteins that are common contaminants of previous tandem affinity purifications (i.e. tubulin and elongation factor 1-alpha). Protein annotation and accession numbers are from the T. brucei 927 database at www.TriTrypDB.org. (DOCX) [file pgen.1008390.s017.docx]

S3 Table : Mass spectrometric identification of PNUTS co-purified proteins in *T. brucei*

**PNUTS**

**WT**

| Accession | Annotation | MW | Score | Peptides | Coverage | PSMs | Score | Peptides | Coverage | PSMs |
| --- | --- | --- | --- | --- | --- | --- | --- | --- | --- | --- |
| Tb927.10.11960 | PNUTS | 35.4 | 711.4 | 21 | 83 | 227 | - | - | - | - |
| Tb927.1.2350 | beta tubulin | 49.7 | 596.3 | 22 | 60 | 185 | - | - | - | - |
| Tb927.1.2360 | alpha tubulin | 49.8 | 461.1 | 24 | 61 | 132 | - | - | - | - |
| Tb927.10.4800 | JBP3 | 65.5 | 354.7 | 27 | 48 | 110 | - | - | - | - |
| Tb927.11.16360 | Wdr82 | 37.9 | 307.9 | 11 | 48 | 80 | - | - | - | - |
| Tb11.v5.1035 | heat shock protein 70 (hsp70), putative | 75.2 | 156.5 | 21 | 40 | 44 | - | - | - | - |
| Tb927.9.15400 | ankyrin-repeat protein, putative | 334.1 | 141.9 | 9 | 5 | 67 | 81.5 | 8 | 6 | 32 |
| Tb927.10.2110 | elongation factor 1-alpha | 49.1 | 140.7 | 7 | 31 | 34 | - | - | - | - |
| Tb927.6.4280 | glyceraldehyde 3-phosphate dehydrogenase, glycosomal | 39 | 94.8 | 6 | 24 | 35 | 484.3 | 3 | 12 | 204 |
| Tb927.2.4950 | hypothetical protein, conserved | 155.8 | 59.6 | 5 | 7 | 25 | 73.7 | 5 | 7 | 32 |
| Tb927.9.4350 | hypothetical protein, conserved | 94 | 73.4 | 2 | 5 | 24 | 40.0 | 4 | 11 | 13 |
| Tb927.6.3750 | heat shock 70 kDa protein, mitochondrial precursor, putative | 71.4 | 84.7 | 11 | 23 | 23 | - | - | - | - |
| Tb927.11.4500 | hypothetical protein, conserved | 101.9 | 52.5 | 5 | 10 | 20 | 94.6 | 5 | 9 | 35 |
| Tb927.11.1130 | calpain-like cysteine peptidase, putative | 613.6 | 54.4 | 8 | 3 | 19 | - | - | - | - |
| Tb927.10.15410 | glycosomal malate dehydrogenase | 33.7 | 50.9 | 3 | 16 | 18 | 59.6 | 4 | 19 | 20 |
| Tb927.6.3220 | hypothetical protein, conserved | 115.6 | 47.4 | 1 | 2 | 18 | - | - | - | - |
| Tb927.2.2650 | hypothetical protein, conserved | 367.5 | 54.6 | 5 | 3 | 17 | 60.4 | 5 | 3 | 17 |
| Tb927.6.2580 | hypothetical protein, conserved | 77.9 | 40.1 | 3 | 6 | 17 | - | - | - | - |
| Tb927.5.1450 | receptor-type adenylate cyclase GRESAG 4, putative | 135.7 | 50.7 | 8 | 13 | 16 | - | - | - | - |
| Tb927.4.3530 | hypothetical protein, conserved | 84.3 | 38.8 | 4 | 10 | 16 | - | - | - | - |
| Tb927.11.2990 | KREPB4 | 46.4 | 33.1 | 1 | 2 | 16 | - | - | - | - |
| Tb927.11.3250 | dynein heavy chain, putative | 530.8 | 42.3 | 12 | 5 | 15 | 36.5 | 9 | 4 | 12 |
| Tb927.8.5580 | N-terminal region of Chorein, a TM vesicle-mediated sorter/Integral peroxisomal membrane peroxin, putative | 526.4 | 40.8 | 10 | 4 | 15 | 24.0 | 6 | 3 | 9 |
| Tb927.10.4200 | hypothetical protein, conserved | 100 | 40.1 | 2 | 4 | 15 | - | - | - | - |
| Tb927.11.1340 | Flagella connector protein 1 | 127.8 | 39.1 | 5 | 10 | 14 | - | - | - | - |
| Tb927.10.13350 | hypothetical protein, conserved | 121.3 | 37.4 | 2 | 4 | 14 | - | - | - | - |
| Tb927.10.5350 | dynein heavy chain, putative | 474.4 | 40.2 | 7 | 4 | 13 | - | - | - | - |
| Tb927.10.5850 | N-terminal region of Chorein, a TM vesicle-mediated sorter, putative | 519.4 | 38.6 | 10 | 4 | 13 | - | - | - | - |
| Tb927.5.3230 | hypothetical protein, conserved | 240.8 | 37.7 | 5 | 4 | 13 | - | - | - | - |
| Tb927.4.620 | hypothetical protein, conserved | 276.2 | 34.5 | 10 | 7 | 13 | - | - | - | - |
| Tb927.10.4170 | hypothetical protein, conserved | 291.2 | 32.5 | 5 | 3 | 13 | 26.3 | 7 | 10 | 13 |
| Tb927.10.9380 | hypothetical protein, conserved | 60 | 31.4 | 3 | 10 | 13 | - | - | - | - |
| Tb927.9.410 | variant surface glycoprotein (VSG, pseudogene), putative | 14.6 | 29.7 | 1 | 13 | 13 | - | - | - | - |
| Tb927.7.710 | heat shock 70 kDa protein, putative | 70.2 | 43.9 | 5 | 14 | 12 | - | - | - | - |
| Tb927.11.12840 | Domain of unknown function (DUF4486), putative | 434.1 | 37.2 | 7 | 4 | 12 | 55.6 | 10 | 4 | 18 |
| Tb927.8.8310 | chaperone protein DnaJ, putative | 51.7 | 36.6 | 2 | 7 | 12 | 55.4 | 3 | 9 | 17 |
| Tb927.7.3160 | Cytoplasmic dynein 1 heavy chain (DYNC1H1), putative | 596.1 | 35.1 | 11 | 5 | 12 | 40.5 | 7 | 2 | 17 |
| Tb11.v5.0251 | phosphatase, putative | 46.6 | 34.0 | 1 | 5 | 12 | - | - | - | - |
| Tb927.4.530 | hypothetical protein, conserved | 55.1 | 33.8 | 2 | 6 | 12 | 25.3 | 2 | 6 | 9 |
| Tb927.9.10140 | hypothetical protein, conserved | 91.7 | 32.6 | 3 | 8 | 12 | - | - | - | - |
| Tb927.11.16890 | Tetratricopeptide-like helical domain containing protein | 228.7 | 32.6 | 9 | 9 | 12 | - | - | - | - |
| Tb927.5.3800 | glutamine hydrolysing (not ammonia-dependent) carbomoyl phosphate synthase, putative | 204 | 35.2 | 6 | 7 | 11 | - | - | - | - |
| Tb927.7.2080 | cap guanylyltransferase-methyltransferase 1 | 116.5 | 32.9 | 5 | 8 | 11 | 25.1 | 3 | 7 | 8 |
| Tb927.8.7990 | Thioredoxin-like/Protein of unknown function (DUF3638)/Protein of unknown function (DUF3645), putative | 550.2 | 32.3 | 7 | 3 | 11 | - | - | - | - |
| Tb927.6.3280 | cyclosome subunit 1, putative | 202 | 32.2 | 2 | 3 | 11 | 65.3 | 3 | 2 | 22 |
| Tb927.10.15570 | transcription factor IIa, putative | 62.7 | 31.4 | 2 | 4 | 11 | - | - | - | - |
| Tb09.v4.0050 | variant surface glycoprotein (VSG), putative | 50.9 | 30.8 | 3 | 8 | 11 | - | - | - | - |
| Tb927.7.4130 | hypothetical protein, conserved | 269.2 | 29.1 | 4 | 3 | 11 | - | - | - | - |
| Tb927.11.14540 | hypothetical protein, conserved | 65 | 24.9 | 3 | 10 | 11 | - | - | - | - |
| Tb927.5.2040 | hypothetical protein, conserved | 36.7 | 24.9 | 6 | 23 | 11 | - | - | - | - |
